# Towards FAIR and federated data ecosystems for interdisciplinary research

**DOI:** 10.1371/journal.pcbi.1013806

**Published:** 2026-02-13

**Authors:** Sebastian Beyvers, Jannis Schlegel, Lukas Brehm, Maria Hansen, Alexander Goesmann, Frank Förster

**Affiliations:** 1 Bioinformatics and Systems Biology, Justus Liebig University Giessen, Giessen, Germany; 2 Bioinformatics Core Facility, Justus Liebig University Giessen, Giessen, Germany; Yale School of Public Health, UNITED STATES OF AMERICA

## Abstract

Scientific data management is at a critical juncture, driven by exponential data growth, increasing cross-domain dependencies, and a severe reproducibility crisis in modern research. Traditional centralized data management approaches are not only struggling with data volume but also fail to address the fragmentation of research results across domains. This hinders scientific reproducibility and cross-domain collaboration and increases concerns about data sovereignty and governance. This article proposes FAIR and federated Data Ecosystems as an improved architectural pattern for future research data ecosystems. It tries to incorporate the latest advancements in decentralized, distributed systems into existing research infrastructure to promote cross-domain collaboration. Based on established patterns from Data Commons, Data Meshes, and Data Spaces, our approach focuses on a layered architecture that consists of governance, data, service, and application layers. With this, it could be possible to preserve domain-specific expertise and control while facilitating data integration through standardized interfaces and semantic enrichment. Key requirements include adaptive metadata management, simplified user interaction, robust security, and transparent data transactions. Our architecture supports compute-to-data as well as data-to-compute paradigms, implementing a decentralized peer-to-peer network that scales horizontally. This article aims to provide both an impulse for the technical architecture as well as concepts for a governance framework so that FAIR and federated Data Ecosystems could enable researchers to build on existing work while maintaining control over their data and computing resources. This could provide a practical path towards an integrated research infrastructure that respects domain autonomy as well as interoperability requirements.

## 1 Introduction

Exponential scientific data growth and increasing cross-domain dependencies create urgent needs for new data management solutions [1]. Next-generation sequencing technologies, for example, have heavily transformed genomic research [2] but also revealed a critical challenge: our current infrastructure struggles to handle not just the volume of data but also the intricate relationships between research outputs across domains. In handling these challenges, traditional solutions like data warehouses, data lakes, and domain-specific repositories are reaching their limits when dealing with large, interconnected datasets with strong sovereignty constraints [3].

This challenge extends beyond storage and processing. The reproducibility crisis in modern research [4,5], worsened by fragmented data management practices, demands immediate action. While initiatives like National Research Data Infrastructure [6], European Open Science Cloud [7], and various Data Commons [8,9] have begun addressing these challenges, they often operate in isolation.

From a technological standpoint, Cloud technologies offer elastic resource allocation [10,11] but can create data sovereignty and privacy challenges. Recent architectural patterns such as Data Commons [8] and Data Spaces [12] demonstrate the potential of distributed approaches aligned with Findable, Accessible, Interoperable, Reusable (FAIR) principles [13]. However, these solutions often rely on centralized control or focus more on domain-specific solutions. This often results in the formation of new silos rather than achieving true cross-domain integration.

We propose FAIR: an architectural pattern that combines the benefits of distributed systems with the reality of existing research infrastructures. This approach integrates established domain-specific repositories with support for intermediate research outputs, including experimental data, analysis workflows, and computational results. By providing both the technical architecture and hints for a governance framework for cross-domain collaboration, FFDE could enable researchers to build upon existing work while maintaining control over their data and computational resources.

This paper argues that the technology for such systems already exists. Federated peer-to-peer systems [14] and ideas for distributed governance models [15] are already in productive use today. But, the fragmented understanding of the requirements and benefits makes it difficult to convince governing bodies and researchers to integrate such systems. We illustrate how combining proven patterns from Data Commons, Data Spaces, and Cloud Computing can create a foundation for future scientific collaboration, one that respects both the sovereignty of individual research groups and the need for easy data integration across domains.

## 2 Drawing from existing architectural patterns

Three architectural patterns have emerged for modern research data management: Data Commons, Data Mesh, and Data Spaces.

The Data Commons pattern [8] (e.g., NIH Data Commons Australian Research Data Commons [16–18]) establishes a centralized, collaborative platform where communities can access, integrate, and analyze data collectively, benefiting from unified standards and shared resources for improved discovery and consistent quality. However, this centralized approach faces two main challenges. First, as data from diverse sources continues to grow, managing and processing these large volumes can strain the central infrastructure, often requiring a significant investment in cloud resources. Second, it is difficult for new organizations to participate in a Data Commons because most are designed for a predefined set of members and the participating organizations may lose control over their data management practices since they must adhere to unified standards and policies established by the central platform.

Another very popular pattern is the the Data Mesh pattern [19,20]. It treats data as a product owned by teams from specific domains, emphasizing decentralized data ownership and federated governance to manage large-scale data across organizations. Although this approach increases interoperability and scalability, it introduces coordination and integration complexities between different domain-owned data products. Each team may implement its own standards, technologies, and governance practices, which makes ensuring consistent data quality and collaboration across the organization challenging. While the domain oriented thinking is clearly a good approach for data management, data meshes were developed for enterprise environments where standardized infrastructure and consistent regulatory frameworks are typical. Research environments present different challenges due to heterogeneous systems and varying institutional policies, which hinders a direct application.

Lastly, Data Spaces [12] are a decentralized framework that enables secure, sovereign data sharing between independent organizations through trust frameworks and granular access controls. This offers benefits such as enhanced data sovereignty and standardized interoperability. Yet, they come with challenges like high implementation costs, since establishing the necessary infrastructure, security measures, and compliance processes requires significant investment and coordination among participants. Complex multi-party governance also poses difficulties, as aligning the interests, policies, and responsibilities of multiple organizations demands robust mechanisms for decision-making and conflict resolution. Adoption with this approach is also challenging due to network effects, because the value of participating in a Data Space increases as more organizations join, making it difficult to achieve critical mass and incentivize early adopters.

Although each architecture offers valuable features, the biggest challenge is combining their complementary strengths to create a unified architecture. FFDEs, therefore, try to selectively build on the strengths of these patterns. From Data Commons, FFDEs adopt standardized metadata schemas for cross-domain interoperability and community curation approaches for data quality assurance. Data Mesh contributes the fundamental principle of domain ownership, which allows participants to maintain control over their data while participating in larger ecosystems, alongside federated governance mechanisms and technological flexibility that accommodate diverse research practices. Data Spaces provide essential sovereignty preservation mechanisms, policy enforcement systems for fine-grained access control, and trust frameworks that enable secure multi-institutional collaboration while maintaining compliance with regulatory requirements.

## 3 FAIR and federated data ecosystems

Modern research increasingly relies on collaboration across different fields. For instance, findings from fluid dynamics can improve weather simulations, which in turn influence agricultural research on resilient plant breeds [21]. As we cannot predict which domains will need to collaborate in the future, research infrastructures must support flexible, cross-domain integration while preserving domain expertise and data sovereignty.

FFDEs represent a vision for addressing these challenges by combining the complementary strengths of Data Commons, Data Mesh, and Data Spaces architectures to extend FAIR principles across organizational boundaries. To successfully implement FFDEs several key requirements must be met which address both technical capabilities and practical usability across research communities. Table 1 outlines the detailed considerations along with some examples for current existing approaches associated with each requirement.

**Table 1:** To enable a scalable, secure, and interoperable domain-agnostic data ecosystem, several key requirements must be addressed. These requirements balance technical capabilities with practical usability while ensuring broad adoption across research communities.

Requirement	Key Considerations	Existing Work
**Governance**	The system must accommodate existing domain-specific governance structures while enabling multiple governance paradigms to coexist effectively. Common baseline rules should be defined for all participating entities, ensuring transparency in governance policies through both human-readable and machine-actionable formats. For privacy protection, policy information disclosure should be limited to those aspects that directly affect public or external access.	The GA4GH Framework [30] sets common standards while allowing diverse implementations, like the European Genome-phenome Archive [31] where independent Data Access Committees manage datasets in shared infrastructures. The GA4GH Beacon API uses tiered access controls that adjust visibility by authentication level, protecting privacy while enabling federated genomic discovery.
**Adaptability of Metadata Management**	The metadata management system should support domain-specific requirements by separating technical metadata from semantic metadata, enabling data owners to maintain full control over their semantics. This separation allows existing data sources to participate without major restructuring. The required technical metadata includes core information about data sharing permissions, conditions, relationships between records, and storage locations. Ideally, the semantic metadata should be integrated using terms from domain-specific ontologies in widely adopted metadata standards. This may necessitate the use of ontology and terminology services to map and transform terms from different ontologies.	Widely adopted standards like Schema.org [32] or Dublin Core [33] enable domain extensions (Bioschemas [34], Darwin Core [35]) that add semantic vocabularies while preserving the base technical framework. Catalog standards like DCAT [27] explicitly separate technical access metadata from domain-specific semantic descriptions. Metadata may also include references to data management plans, which describe how researchers create and use their data and the rules they follow.
**Simplifying User Interaction**	To achieve widespread adoption, user interaction must be as easy as possible through the integration of existing repositories and widely accepted interfaces. Technical adapters play a crucial role by transforming various existing data exchange formats into unified, well-established standards, ensuring ease of use and compliance with FAIR principles.	Research institutions use technical adapters to automatically convert heterogeneous data formats (CSV exports, SQL databases, lab software outputs) into standardized metadata schemas and OAI-PMH [28] protocols, allowing scientists to query all datasets through a single search interface.
**Data Sovereignty and Security Measures**	Maximizing data sovereignty requires implementing robust access control systems with advanced security measures, including encryption and continuous monitoring of data access as well as dependencies. Multi-layered protection systems should prevent unauthorized access and data breaches while ensuring compliance with regulations like GDPR. Data providers should retain final decision authority, supported by decentralized, peer-to-peer data transfer mechanisms that minimize reliance on central authorities.	GDPR now requires all providers of sensitive data to implement robust access control, encryption and data protection standards.
**Transparency in Data Transactions**	The system should provide data owners and consumers with clear visibility into data access, usage, and compliance with privacy and ethical standards like CARE [36] or the Nagoya Protocol [37]. Everyone should have a comprehensive understanding of data handling processes, supported by robust authentication systems that enable identity verification for all stakeholders involved in data transactions.	Dataspaces may include extensive compliance monitoring and audit trails. This is usually achieved through an event database and/or event sourcing.

One of the most important decisions is the network protocol, which determines how participants interact with each other. For the greatest autonomy, we believe that a fully decentralized, peer-to-peer network is the best starting point for data and metadata exchange. In this type of network, no single entity controls the infrastructure. While these have been difficult to implement in the past, large infrastructures like IPFS [22] now make them accessible through libraries like libp2p. Peer-to-Peer (P2P) architectures are particularly suited for scientific data exchange as they enable direct connections between research institutions regardless of their network configurations, automatically handling NAT traversal and firewall constraints that often isolate academic networks, while providing resilient data access even when individual nodes or entire institutions temporarily go offline. Modern P2P technologies typically include distributed hash tables like Kademlia [23] for discovering peers and datasets, enabling decentralized data discovery.

The second challenge is building trust between participants. For this it is crucial to reliably identify users, institutions and even services. To implement this we can adopt the ideas from Data Spaces that describe a trust framework in which trust is derived by a chain of cryptographic certificates and signatures that prove the identity of a participant. Rather than building new identity systems, we can leverage existing federated infrastructure like eduGAIN [24], which already connects thousands of research institutions worldwide. This approach builds on decades of investment in academic identity federation while extending trust relationships to include not just users but also institutions and computational services.

The third component addresses data discovery and access governance through federated catalogs. Standardized protocols like Open Digital Rights Language (ODRL) [25] or Data Use Conditions [26] communicate governance policies and access terms, while DCAT-based [27] semantic descriptions enable automated discovery of available datasets, services and conditions. Each participant maintains sovereignty over their data models and quality standards, exposing only the minimal technical metadata necessary for discovery and access through standardized interfaces.

Together, these three components create a data plane that handles networking, identity, trust, and discovery without requiring participants to sacrifice their existing infrastructure or domain expertise. This data plane is the technical foundation for enabling higher-level services: federated search engines that span multiple institutions, analytical workflows accessing cross-domain datasets, web portals that provide familiar interfaces to distributed resources, and AI systems that can identify patterns across previously isolated data silos.

**Fig 1:**
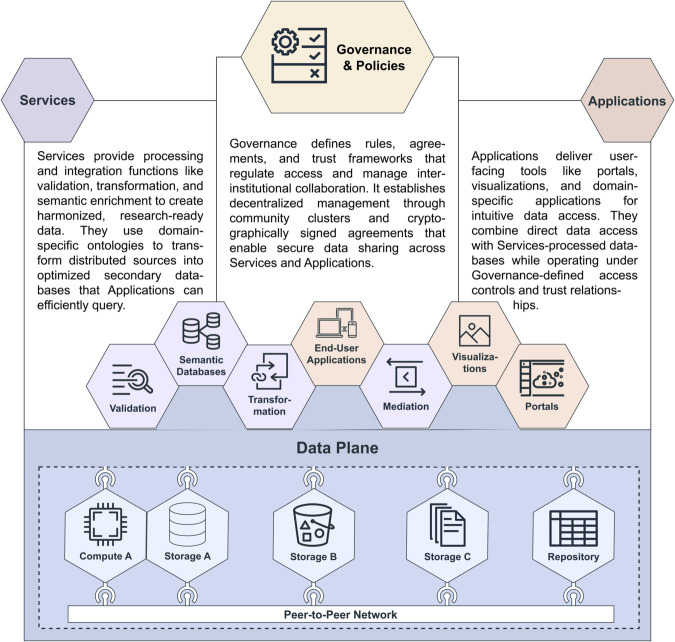
Schematic overview of the proposed architecture, illustrating the interplay between data services, application services, governance structures, and the underlying data plane. Storage and compute nodes are interconnected with each other through an internal peer-to-peer network; connections to existing systems are established by adapters. All services and applications build on top of the dataplane and connect via standardized interfaces like DCAT.

The data plane can thus serve as the foundation for three additional architectural planes that complete the FFDE ecosystem (see Fig 1). The Governance and Access Control Plane implements decentralized governance through community clusters and federated identity management. The Service Plane transforms distributed data sources into integrated secondary resources through automated processes and semantic enrichment, providing a transformed view into the existing data. Finally, the Application Plane constructs user-facing interfaces that abstract the distributed architecture complexity, allowing researchers to focus on scientific questions rather than system management. This plane can also include traditional repositories and other user-facing applications. A more detailed description of these planes is found in Table 2.

**Table 2:** Architectural components of the decentralized research data infrastructure framework. The four-plane architecture enables distributed data governance, peer-to-peer data management, semantic data integration, and user-accessible research applications while maintaining institutional autonomy and data sovereignty. Examples of existing technologies and research application that could be used to implement this plane.

Architectural Component	Details	Example technologies
**Governance and Access Control Plane**	Implements decentralized governance through community clusters organized by institutional type, research domain, or geographic region. Uses standardized data sharing agreements, federated identity management (e.g., eduGAIN [24]), attribute-based access control policies, and cryptographically signed Memoranda of Understanding. Establishes inter-institutional trust relationships while maintaining comprehensive audit trails and fast-track approval processes for trusted partners.	This component could leverage eduGAIN [24] for federated identity management across research institutions, with access control implemented Role-Based Access Control. Institutional trust relationships could be established using GÉANT Trust frameworks and Research Data Alliance (RDA) [38] governance standards, while audit trails are maintained through traditional event systems like MQTT.
**Data Plane**	Operates a decentralized peer-to-peer network with Data Locations (sovereign storage units) and Compute Nodes (processing resources with standardized adapters). Supports both compute-to-data and data-to-compute paradigms, optimizing distribution based on processing capabilities, bandwidth, privacy constraints, and geographical location. Scales horizontally while maintaining governance through federated trust domains and automatic relationship mapping between datasets.	This component could be implemented using iRODS [39] for distributed data management across institutions. Compute-to-data paradigms could leverage Galaxy workflows [40] and HTCondor [41] for distributed processing, while DataONE [42] provides federated metadata cataloging and automatic dataset relationship mapping. The peer-to-peer network could utilize IPFS [22] for decentralized storage with ESGF (Earth System Grid Federation) [43] patterns for domain-specific data federation.
**Service Plane**	Transforms distributed data sources into integrated secondary resources through automated transformation processes and semantic enrichment. Uses domain-specific ontologies (e.g., Gene Ontology [44], Disease Ontology [45]) to harmonize data across sources. Creates specialized secondary databases optimized for research queries while maintaining eventual consistency with primary distributed sources through automated synchronization.	This component could utilize Nextflow [46] and Snakemake [47] for automated scientific workflow orchestration and transformation processes, with Galaxy providing user-friendly data transformation interfaces. Semantic enrichment could be implemented through Apache Jena [48] for ontology processing. Secondary database creation could leverage DataVerse [49] for research data repositories and eventual consistency maintained through Apache Kafka streaming between distributed sources.
**Application Plane**	Constructs user-facing interfaces by combining direct access to Data Plane raw sources with optimized Service Plane databases. Implements diverse interfaces, including web dashboards, data portals, RESTful APIs, command-line tools, and plugin frameworks. Abstracts the distributed architecture complexity, allowing researchers to focus on scientific questions rather than system management while providing access to cross-domain datasets.	This component could be implemented using CKAN [29] and DataVerse [49] for research data portals, with JupyterHub providing interactive computational interfaces and R Shiny for scientific dashboards. RESTful APIs could be built using DataCite and ORCID APIs for researcher identity and data citation, while Galaxy Tool Shed provides plugin framework capabilities for extensible functionality. Cross-domain dataset discovery could utilize DataONE search interfaces and OpenAIRE Explore for federated research data access.

The important point is that these plains can be implemented today using existing technologies. The challenge isn’t technical innovation but organizational coordination, establishing the governance frameworks that allow institutions to connect their existing infrastructure into a federated research ecosystem. What remains is filling the gaps and coordinating these technologies through unified governance. Successful implementations must therefore integrate with established repository standards such as OAI-PMH [28], CKAN [29], and DCAT [27]. Additionally, they should expose these same standards to enable other implementations to harvest their data, ensuring bidirectional interoperability across the ecosystem.

The benefits compound with scale. Each new institution that joins brings not just data but also domain expertise, computational resources, and research communities. In an era of AI-driven discovery, where machine learning models can identify patterns across seemingly unrelated datasets, the value of cross-domain data access becomes exponential. Knowledge graphs can automatically link concepts across fields, while automated reasoning systems can suggest novel research directions, but only if the data is accessible.

### 3.1 The researchers perspective

Consider research investigating plant flowering responses to temperature changes, requiring integration of climate records with genomics data across institutions. In the current world, finding and gathering the necessary data is mostly a manual, labor-intensive process. However, with the widespread adoption of FFDEs, this process could be much easier and faster.

Finding suitable data and access requirements would become the researcher’s starting point. Cross-domain discovery operates through federated catalogs that aggregate metadata from participating institutions while maintaining distributed control. These catalogs are built on widely adopted metadata standards such as Schema.org [32] or Dublin Core [33] that serve as the base metadata layer for all participating nodes. These can be extended with domain-specific metadata schemas and profiles like CF standards for climate data or BioSchemas [34] for genomics datasets, all synchronized through the P2P protocol to enable automated linking and discovery. Crucially, the metadata includes machine-readable rights and duties expressed in standards like ODRL, allowing researchers to immediately understand what permissions they need, what usage restrictions apply, and what obligations they must fulfill to access each dataset. Moreover, this may only be the first step in the discovery process. If the domain already has an established ontology and/or metadata format, more detailed metadata files can be retrieved in this highly specialized, domain-specific form.

Locating distributed data follows once relevant datasets and their access requirements are identified. The federated ecosystem relies on content-addressed storage systems, which ensure data integrity and enable efficient distribution. These systems are proven technology that has been in use for decades (e.g., BitTorrent), allowing researchers to locate exact copies of datasets across multiple institutions without concerns about data corruption or version mismatches.

Accessing data through institutional networks becomes possible through a unified peer-to-peer networking protocol that connects all participating nodes regardless of their current network setup. Genomics institutions might implement specialized interfaces following established specifications like GA4GH standards [30], while climate research centers could provide domain-optimized protocols for temporal environmental datasets, yet all participate in the same federated network through the underlying P2P synchronization mechanism. Processing data with portable workflows and tasks eliminates the need for large-scale data movement. Computational workflows defined using portable standards like Common Workflow Language can execute across federated computing resources, allowing researchers to process distributed datasets through familiar analytical platforms. Individual researchers can directly join the network with their own computational resources, creating a resilient distributed system that requires no central coordinating instance or single point of failure.

Maintaining compliance and audit trails operates through existing academic federation systems, allowing researchers to authenticate using their institutional credentials while fine-grained policy engines enforce the machine-readable data usage agreements discovered during the metadata phase. Immutable audit logs capture every data access and usage event across the distributed network, providing transparent accountability and enabling compliance verification while supporting reproducibility requirements. The institutional benefits extend beyond technical efficiency: participating organizations retain complete control over their data while gaining access to previously siloed resources, reducing infrastructure costs through shared computational resources, and enhancing their research impact through expanded collaboration opportunities. For researchers, this architecture eliminates the traditional barriers between domains, making discovery of relevant datasets across institutions as straightforward as searching within a single repository, while portable workflows ensure that analyses remain reproducible and shareable regardless of where they execute. The resulting research environment preserves the specialized expertise and governance structures that different domains require while creating new opportunities for interdisciplinary discovery, ultimately enabling scientific investigations that would be impossible within traditional institutional boundaries.

## 4 Discussion and conclusion

The architectural components outlined in this paper show that the technical foundation for FFDEs already exists. Production systems already handle distributed data at petabyte scale. Identity federations connect thousands of institutions. Governance frameworks from existing initiatives provide working templates for federated control. When you combine these mature technologies with new AI capabilities, knowledge graphs, and automated reasoning, the opportunities for cross-domain scientific discovery grow rapidly.

The potential benefits of such an architecture extends beyond technical efficiency. By preserving institutional sovereignty while enabling collaboration, this approach addresses the fundamental tension between data control and data sharing. It promises reduced infrastructure costs through resource pooling, better data quality through semantic enrichment, and faster discovery through cross-domain integration. Today’s breakthroughs increasingly happen at disciplinary boundaries. Climate data informs epidemiology, genomics advances materials science. In this context, widespread data integration becomes increasingly valuable.

The framework we’ve proposed is deliberately flexible, accommodating everything from fully open science to heavily regulated closed research environments. It also can support many forms of data management, from rapidly changing “hot” datasets that are currently researched as well as data publications. In theory it could also accommodate textual publications, but it is not realistic to do so in the near future.

However, realizing this vision involves navigating significant non-technical challenges. Establishing federated governance structures requires reconciling different institutional policies, regulatory frameworks, and cultural practices around data sharing. Legal complexities arise from varying data protection regulations across jurisdictions, intellectual property concerns, and liability questions in collaborative research. The heterogeneity of existing systems, each with established workflows, user communities, and investment, creates substantial integration challenges beyond mere technical compatibility. Community acceptance is critical for federated data sharing success. Organizations like the Elixir network are already developing standards, providing valuable collaboration opportunities. Interoperating with existing repositories reduces adoption barriers without disrupting current workflows. This approach rapidly aggregates datasets using existing infrastructure, avoiding major hardware investments. Demonstrating immediate value with minimal investment builds momentum and community trust.

Another adoption barrier involves metadata harmonization in domains with established standards. Our multi-tiered approach requires maintaining multiple metadata representations: broad summaries in standards like Schema.org for cross-domain discovery alongside rich domain-specific metadata for specialized use. This dual maintenance burden may deter communities that have invested heavily in their metadata ecosystems. Successfully bridging domains will require robust terminology and ontology services to map concepts between specialized vocabularies. While such services exist, integrating them into federated workflows and convincing communities of their value represents another coordination challenge beyond technical implementation.

Perhaps most critically, the benefits of federated ecosystems must be effectively communicated to diverse stakeholders. Researchers need assurance that federation enhances rather than complicates their work. Institutions require evidence that sovereignty and security are maintained. Funding bodies seek demonstration of tangible returns on infrastructure investment. Without clear articulation of these benefits and honest acknowledgment of the challenges, even technically superior solutions may face adoption barriers.

The transition from isolated repositories to federated ecosystems represents both a technical evolution and a cultural shift in how scientific communities approach data. While the technology stands ready, success will ultimately depend on building trust, demonstrating value, and creating governance frameworks that balance openness with control. The growing urgency of global challenges, from climate change to pandemic response, underscores the importance of this transition. Each day of continued fragmentation represents missed opportunities for discovery and innovation that our interconnected world can no longer afford.
